# The association between accessing dental services and nonventilator hospital-acquired pneumonia among 2019 Medicaid beneficiaries

**DOI:** 10.1017/ice.2022.163

**Published:** 2023-06

**Authors:** Dian Baker, Karen K. Giuliano, Madhuli Thakkar-Samtani, Frank A. Scannapieco, Michael Glick, Marcos I. Restrepo, Lisa J. Heaton, Julie Frantsve-Hawley

**Affiliations:** 1 School of Nursing, California State University–Sacramento, Sacramento, California; 2 Elaine Marieb Center for Nursing and Engineering Innovation, Institute for Applied Life Sciences & Elaine Marieb College of Nursing, University of Massachusetts–Amherst, Amherst, Massachusetts; 3 Biostatistician, Analytics and Evaluation, CareQuest Institute for Oral Health, Boston, Massachusetts; 4 Department of Oral Biology, School of Dental Medicine, University at Buffalo School of Dental Medicine, Buffalo, New York; 5 Center for Integrative Global Oral Health, University of Pennsylvania School of Dental Medicine, Philadelphia, Pennsylvania; 6 Division of Pulmonary and Critical Care Medicine, Department of Medicine, South Texas Veterans’ Health Care System, San Antonio, Texas; 7 Joe R. & Teresa Lozano Long School of Medicine The University of Texas Health–San Antonio, San Antonio, Texas; 8 Analytics and Evaluation, CareQuest Institute for Oral Health, Boston, Massachusetts; 9 TAG Oral Care Center for Excellence, Chicago, Illinois

## Abstract

In this 2019 cross-sectional study, we analyzed hospital records for Medicaid beneficiaries who acquired nonventilator hospital-acquired pneumonia. The results suggest that preventive dental treatment in the 12 months prior or periodontal therapy in the 6 months prior to a hospitalization is associated with a reduced risk of NVHAP.

Hospital-acquired pneumonia (HAP) is the number one cause of hospital-acquired infections, and nonventilator hospital-acquired pneumonia (NVHAP) represents 60% of cases.^1^ NVHAP occurs in ∼1 in 100 hospitalized patients and has an associated crude mortality of 15%–30%.^2–4^ NVHAP is associated with increases in antibiotic usage, intensive care unit utilization rates, and readmission rates, and it is the most common pathway to sepsis.^4^ Despite the known harm, much less is understood about how to prevent NVHAP.

HAP results from aspiration of microbes from the upper airway following admission to a hospital. The mouth harbors a diverse assortment of microbes, which become established on surfaces such as the teeth, tongue, and mucosa. The teeth, in particular, have the capacity to support millions of microbes as biofilms, and these biofilms can expand rapidly in the absence of effective oral hygiene. The lung has a large surface area exposed to the environment and is directly influenced by microflora originating in the oropharyngeal cavity.^5,6^ Aspiration of oral microbes can easily occur during hospitalization due to impaired clearance of oropharyngeal secretions from diminished salivary flow and difficulties in swallowing.^6^ Inoculation of the lung by aspirated pathogens derived from dental biofilm can also incite an inflammatory response increasing the risk of NVHAP.^6^ Biologically active mediators are often elevated in the secretions of patients with poor oral hygiene and periodontal disease, which can directly induce inflammation in the lung when aspirated.^5,6^ Thus, effective oral hygiene should reduce the numbers of microbes that could be aspirated, and thus would reduce the risk of NVHAP.

During the last 20 years, research has demonstrated that oral health and overall health are inherently linked.^7^ Emerging evidence supports that professional oral care prior to surgery for cardiovascular disease and cancer can result in a reduction of postoperative pneumonia.^8,9^ In the acute-care setting, there is an emerging body of evidence on the role of oral care for NVHAP prevention.^4^ Despite this evidence, research on the relationship of preventive dental services prior to hospitalization and NVHAP is limited. We sought to determine whether utilization of dental services prior to hospitalization is associated with a decreased risk of NVHAP.

## Design

Multistate 2019 data obtained from the IBM Watson MarketScan Medicaid Database were used to determine incidence of NVHAP. The IBM MarketScan Medicaid Research Databases captures deidentified person-specific information on medical and dental clinical utilization, administrative claims, and enrollment across both inpatient and outpatient services from 13 deidentified states. All Medicaid beneficiaries who were admitted to a hospital at some point in 2019 and had no missing inpatient claims data were included. The Western Institutional Review Board approved this study (no. ANP0008, May 2018).

NVHAP cases were first identified using related, not present on admission, codes in the *International Classification of Diseases, Tenth Revision, Clinical Modification* (ICD-10-CM) and then were verified by the secondary diagnosis-related group (DRG) code for pneumonia on the second or later day of hospital admission. In beneficiaries with NVHAP, using dental claims data and Current Dental Terminology (CDT) codes, 2 types of dental services were identified: (1) number of dental diagnostic, prophylaxis, or preventive visits, and (2) scaling and root planing (SRP) services for periodontal disease received in the 2 years prior to their 2019 hospitalization.

First, descriptive statistics and χ^2^ analyses were used to describe relationships. Next, several potential confounding factors were controlled: comorbidities, age, sex, race, surgical procedures requiring intubation during inpatient stay, prior hospitalization, history of smoking, and complete edentulism status (based on current dental terminology [CDT] codes for complete dentures), and hospital length of stay. Individual comorbidities were identified to create the Elixhauser comorbidity index score based on ICD-10-CM diagnosis codes. After adjusting for these confounding factors, a multiple linear regression model was used to determine whether the utilization of dental services prior to hospitalization was significantly associated with NVHAP. Each unique beneficiary was the unit of analysis. All analyses were performed using Stata version 16.0 software (StataCorp, College Station, TX).

## Results

Table 1 provides a summary of the sample demographics and relevant characteristics by incidence of NVHAP. Overall, 13.7% (N = 13,866) of the 1,012,025 Medicaid beneficiaries admitted to the hospital for ≥2 days in 2019 were diagnosed with NVHAP, with incidence rates of 1.95 per 1,000 patient days and 121.9 per 100,000 Medicaid participants. NVHAP occurred in across sociodemographic groups.

**Table 1.** Descriptive Analysis of Patients Enrolled in Medicaid Hospitalized for Inpatient Services in 2019 by Incidence of Nonventilator Hospital-Acquired Pneumonia (NVHAP)

Variable	Overall (N=1,012,025), No. (%)	No NVHAP Diagnosis (n=998,159), No. (%)	NVHAP Diagnosis (n=13,866), No. (%)
**Utilization of periodontal services**			
None or >2 y ago	993,202(98.1)	98.10	97.90
<6 mo ago	7,059 (0.7)	0.70	0.60
6 mo to 1 y ago	4,395 (0.4)	0.40	0.50
1–2 y ago	7,369 (0.7)	0.70	1.00
**Past preventive dental visit**			
None	831160 (82.1)	82.10	84.10
At least 1 preventive dental visit in the past year	180,865 (17.8)	17.90	16.00
**Age**			
0–20 y	359601 (35.5)	35.90	10.20
21–64 y	521,583 (51.5)	51.40	63.90
≥65 y	130,841 (12.9)	12.80	26.00
**Sex**			
Male	364,707 (36.0)	35.90	48.20
Female	647,257 (63.9)	64.10	51.90
**Race**			
White	504,227 (56.2)	56.10	61.50
African American	310,037 (34.6)	34.60	33.20
Hispanic	47,077 (5.3)	5.30	2.60
Other	35,845 (4.0)	4.00	2.80
**Controlling factors**			
**Had a surgical procedure requiring intubation during inpatient stay**			
No	995262 (98.3)	98.50	85.60
Yes	16,763 (1.7)	1.50	14.40
**Prior hospitalization in a year**			
No	592,701 (58.6)	58.90	33.90
Yes	419,324 (41.4)	41.10	66.10
**History of smoking**			
No	787,363 (77.8)	86	76
Yes	224,662 (22.2)	14	24
**Length of inpatient stay**	5.5 ± 10.5	5.3 ± 9.9	19.8 ± 25.9
**Complete edentulism**			
No	987,176 (97.5)	97.60	94.40
Yes	24,849 (2.5)	2.40	5.70
**Elixhauser comorbidity score**			
No comorbidities	448,092 (44.3)	44.70	16.90
1 comorbidity	108,862 (10.8)	10.80	5.40
2 comorbidities	89,240 (8.8)	8.90	6.60
3 or more comorbidities	365,831 (36.2)	35.70	71.20

**Fig. 1.**
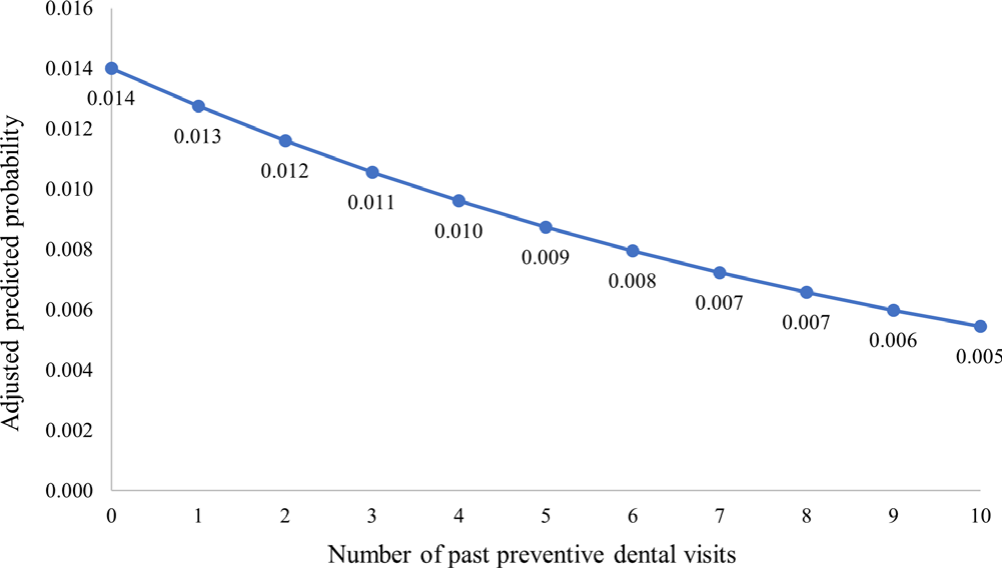
Adjusted predicted probability of acquiring NVHAP.

Predicted probability of diagnosis of NVHAP decreased with each additional preventive dental visit 1 year prior to hospitalization (Fig. 1). In our adjusted model, beneficiaries who had at least 1 preventive dental visit within a year of hospitalization were 10% less likely to get NVHAP (adjusted odds ratio [aOR], 0.90; 95% confidence interval [CI], 0.86–0.95; *P* < .001), compared to beneficiaries with no preventive dental visits or visits >1 year prior to hospitalization.

Additionally, use of periodontal services (SRP) <6 months prior to hospitalization decreased the odds of NVHAP diagnosis by 30% (aOR, 0.70; 95% C, 0.56–0.89; *P* = .003). Receipt of SRP 6–12 months (aOR, 0.85; 95% CI, 0.66–1.08; *P* = .175) or 1–2 years prior to hospitalization (aOR, 0.96; 95% CI, 0.80–1.15; *P* = .0.659) showed no impact on NVHAP incidence (Table 1).

## Discussion

Medicaid beneficiaries carry a risk of NVHAP upon admission to acute-care hospitals and all sociodemographic groups were affected by NVHAP. Medicaid beneficiaries had a higher incidence of NVHAP (1.95 per 1,000 patient days) compared to a Veterans’ Health Administration (VA) large data-set analysis of NVHAP. For veterans (2015–2020), the NVHAP rate was 1.26 cases per 1,000 hospitalized days.^10^ The VA study authors compelled hospitals to implement universal prevention for NVHAP.^10^ To the best of our knowledge, this is the first study to demonstrate a relationship between outpatient preventive dental and periodontal treatment decreased NVHAP during inpatient hospitalization. This finding is consistent with prior research focused on inpatient oral healthcare during acute-care hospitalizations and NVHAP prevention.^4^ These results are also consistent with prior research that presurgical dental care can reduce the incidence of postoperative pneumonia in patients undergoing cancer or cardiovascular surgery.^8,9^ The results of the current study suggest that good oral health that includes receipt of dental health services prior to hospitalization may be an additional strategy for NVHAP prevention during inpatient hospitalization.

This study had several limitations. Dental utilization was used as a proxy for good oral health. In some cases, however, extensive use of dental services, such as SRP, may reflect general poor oral health that is under treatment, rather than treatment to maintain good oral health. Conversely, dental appointments may represent a surrogate marker for an individual’s attention to their overall health, thus decreasing their NVHAP risk. The use of administrative claims data and billing codes for research is also a limitation because variable accuracy could not be confirmed. For example, in this research, dental diagnoses were often inferred through treatments provided rather than defined specifically in claims data. Results from Medicaid beneficiaries’ data may not be generalizable to other insurance systems or in states where dental services are not included as a Medicaid benefit.

A Medicaid beneficiary receiving preventive dental treatment in the year prior, or periodontal therapy in the 6 months prior to a hospitalization, is associated with a reduced risk of NVHAP. These findings support the emerging body of evidence on importance of oral health for NVHAP prevention.
